# Development, Optimization, and Physicochemical Characterization of Fluorescent Carbon Dot‐ *Ginkgo biloba* Extract Co‐Loaded Niosomes for Future Neurological Disease‐Relevant Evaluation

**DOI:** 10.1002/cns.71230

**Published:** 2026-11-01

**Authors:** Yasaman Mohammadi, Mohammad Saied Salehi, Ehsan Heidari, Mina Ebrahimi, Nahid Ashjazadeh

**Affiliations:** ^1^ Clinical Neurology Research Center of Shiraz University of Medical Sciences Shiraz Iran; ^2^ School of Graduate Studies, Joint Ph.D. Program in Biomedical Engineering Rutgers, The State University of New Jersey Newark New Jersey USA; ^3^ Department of Pharmacology and Toxicology, School of Pharmacy Zanjan University of Medical Sciences Zanjan Iran

**Keywords:** Alzheimer's disease, bioavailability, CDOTs, drug delivery, *Ginkgo biloba*, niosomes

## Abstract

**Background:**

Alzheimer's disease (AD) is one of the major neurodegenerative diseases that affect the human brain and lead to memory loss. The present approaches to disease management have been relatively poor; the therapies provided do not arrest the disease process. This study aimed to develop and optimize dual‐loaded niosomes containing *Ginkgo biloba* extract and CDOTs and to evaluate their physicochemical properties, formulation‐level fluorescence, in vitro release, and preliminary cytocompatibility. The study did not assess blood–brain barrier transport, neuronal effects, Alzheimer's disease‐specific mechanisms, or in vivo therapeutic efficacy.

**Results:**

Niosomes were prepared based on non‐ionic surfactants and cholesterol for the encapsulation of Ginkgo and CDOTs. The response surface methodology (RSM) was used to optimize niosomal formulations through a Box–Behnken design. The formulations were evaluated for vesicle size, entrapment efficiency, and release profile. Field Emission Scanning Electron Microscopy (FESEM) research indicated that the average diameters of the free niosomes and CDOT@Ginkgo@Niosomes were 69.56 nm and 72.19 nm, respectively. HFF cell viability remained high at lower concentrations, whereas significant reductions in viability were observed at 10 and 50 μg/mL after 48 h, indicating concentration‐dependent cytocompatibility. The formulation exhibited acceptable preliminary cytocompatibility at lower concentrations, whereas reduced viability was observed at higher concentrations. For both CDOT and Ginkgo, CDOT@Ginkgo@Niosomes showed a delayed release profile over 72 h, with near‐complete release observed at the final time point.

**Conclusions:**

CDOT@Ginkgo@Niosomes showed successful co‐loading, preserved formulation‐level fluorescence, delayed release, and acceptable cytocompatibility at lower concentrations in HFF cells. These findings support further formulation optimization and subsequent evaluation in disease‐relevant neuronal, blood–brain barrier, and in vivo models. The present results do not demonstrate neuroprotection, diagnostic performance, blood–brain barrier transport, or the prevention or treatment of neurodegenerative disease.

## Graphical abstract

Carbon dot– *Ginkgo biloba* –loaded niosomal nanovesicles are fabricated via rotary evaporation, probe sonication, and centrifugation to co‐deliver natural neuroprotective agents to the brain. The optimized system shows high encapsulation, sustained release, and excellent biocompatibility, suggesting a promising nano‐vesicular platform for Alzheimer's disease therapy.

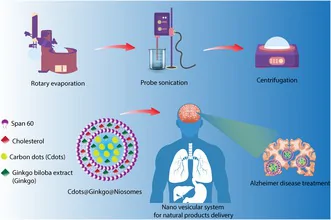

**Abbreviations**
AD: Alzheimer's disease
AFM: atomic force microscopy
BBB: blood–brain barrier
CDOT: carbon dots
DLS: dynamic light scattering
DMEM: Dulbecco's Modified Eagle Medium
DMSO: dimethyl sulfoxide
DSC: differential scanning calorimetry
EE: entrapment efficiency
FTIR: Fourier transform infrared spectroscopy
HFF: human foreskin fibroblast
HOMO: highest occupied molecular orbital
LUMO: lowest unoccupied molecular orbital
MTT: 3‐(4,5‐dimethylthiazol‐2‐yl)‐2,5‐diphenyltetrazolium Bromide (commonly used in cell viability assays)
NP: nanoparticle
OMA: optical multi analysis
PBS: phosphate‐buffered saline
PDI: polydispersity index
QY: quantum yield
RCT: randomized controlled trial
RSM: response surface methodology
SDS: sodium dodecyl sulfate
SEM: scanning electron microscopy
UV: ultraviolet

## 1 Introduction

Alzheimer's disease (AD) is a type of dementia that affects older people and involves loss of memory, impaired intellect, and the ability to carry out daily activities. Alzheimer's disease is the type of dementia most often encountered in clinical practice; it is said to affect between 60% and 80% of dementia cases. The neuropathological changes in AD are represented by extracellular and intracellular amyloid deposits, especially beta‐amyloid, and neurofibrillary tangles mainly composed of the hyperphosphorylated form of the microtubule‐associated protein tau, which result in neuronal death and brain shrinkage [1, 2]. A number of investigations carried out in the past few years have shed light on oxidative stress and inflammation as key players in AD development. These processes are believed to contribute to the development of amyloid plaques and neurofibrillary tangles, which lead to neuronal death and synaptic dysfunction [3, 4]. Given the multifactorial nature of Alzheimer's disease, natural bioactive compounds capable of modulating multiple disease‐associated proteins and signaling pathways have attracted increasing research interest [5].

*Ginkgo biloba* extracts contain flavonoid glycosides and terpene lactones among their principal bioactive constituent classes [6]. Preclinical studies using defined Ginkgo preparations have reported anti‐inflammatory effects in LPS‐activated primary microglial cells and antioxidant effects in APPsw‐overexpressing Neuro 2A cells, respectively [7, 8]. Clinical studies have also evaluated the standardized extract EGb 761 in cognitive disorders, including Alzheimer's disease and multi‐infarct dementia [9]. However, these findings cannot be extrapolated to the dual‐loaded niosomal formulation developed in the present study because extract composition, formulation characteristics, and experimental context may substantially influence biological effects [6]. No neuronal, neuroprotective, or Alzheimer's disease‐specific efficacy experiments were performed in the present study.

In the context of AD, appropriately engineered niosomal formulations, particularly mucoadhesive intranasal systems, have shown promise for enhancing the brain delivery of encapsulated therapeutic agents by improving formulation stability and nasal residence [10] and by facilitating transport through the olfactory and trigeminal pathways, thereby partially circumventing the restrictive BBB [11, 12]. In an AD‐relevant study, intranasally administered niosomes co‐loaded with rivastigmine and N‐acetylcysteine exhibited greater brain distribution than the corresponding non‐niosomal formulations, providing direct preclinical support for this delivery strategy [13]. Such systems may consequently improve drug exposure in the central nervous system; however, the efficiency and regional distribution of brain delivery depend on the administration route, physicochemical properties, surface functionalization, and, where applicable, the incorporation of targeting ligands [12, 14].

Niosomes are vesicular structures constructed from non‐ionic surfactants and cholesterol. Thus, they are characterized by stability, non‐toxicity, biocompatibility, and capacity to stabilize both hydrophilic and lipophilic active components, protect the latter from degradation, and increase their bioavailability [15, 16]. Specifically engineered niosomal systems have been investigated for brain‐directed delivery in AD‐related research [17, 18, 19].

Brain‐directed delivery by niosomes is strongly influenced by formulation design and the route of administration. Chitosan glutamate‐coated niosomes have demonstrated mucin‐interacting and mucoadhesive properties that support their potential application as intranasal nose‐to‐brain delivery systems [10]. More directly, intranasally administered bifunctional niosomes co‐loaded with rivastigmine and N‐acetylcysteine exhibited a favorable pharmacokinetic profile and preferential brain distribution relative to other organs in an AD‐oriented preclinical study [13]. Surface‐modified olanzapine niosomes have similarly demonstrated brain‐targeting potential following intranasal administration in rats, further highlighting the importance of surface engineering in CNS delivery [14]. These findings are consistent with evidence that intranasally administered therapeutics may access the CNS through olfactory and trigeminal pathways, thereby reducing their dependence on transvascular passage across the BBB [11]. Collectively, these studies support the potential of appropriately engineered niosomes for brain‐directed delivery and provide a scientific rationale for future evaluation of BBB transport, neuronal uptake, and biodistribution of the present CDOT@Ginkgo formulation.

The combination of *Ginkgo biloba* extract with its neuroprotective flavonoids and terpenoids shows effectiveness in cognitive function enhancement through its potent antioxidant and anti‐inflammatory properties, according to previous studies [6, 7, 8, 20]. When using citric acid and urea in microwave‐assisted synthesis, highly luminous CDOTs are produced effectively [15]. Interestingly, we observed a significant increase in the quantum yields, moving from 24.11% to 54.75% as we carefully adjusted the ratio of urea. These nanoparticles are characterized by a variety of surface functional groups, including hydroxyl, carbonyl, and amine groups, which significantly enhance their water solubility [15, 16, 21] and may support controlled drug delivery [22].

The incorporation of CDOTs preserved detectable fluorescence at the formulation level, which may support future tracking studies after appropriate cellular and in vivo validation.

The co‐loading of *Ginkgo biloba* extract and fluorescent CDOTs provides a promising combination of a bioactive natural extract and formulation‐level optical functionality. Fluorescent carbon‐dot hybrid systems have previously been investigated for intracellular imaging, supporting the future evaluation of the preserved fluorescence of the present formulation for biological imaging or tracking applications [22]. *Ginkgo biloba* extract has also demonstrated antioxidant and amyloid‐β‐modulating effects in APPsw‐overexpressing Neuro 2A cells [8]; however, the neuroprotective potential of the present CDOT@Ginkgo niosomal formulation requires confirmation in disease‐relevant neuronal and in vivo models.

The present study aimed to establish the physicochemical feasibility of a dual‐loaded niosomal platform combining *Ginkgo biloba* extract as a bioactive payload with fluorescent carbon dots (CDOTs) as an intrinsic optical component. The formulation was developed and optimized to achieve nanoscale vesicles with suitable colloidal characteristics, effective co‐entrapment of both components, preservation of CDOT fluorescence following incorporation, and controlled release of the loaded materials. Particle size, polydispersity index, zeta potential, morphology, apparent entrapment efficiency, formulation‐level fluorescence, in vitro release behavior, and concentration‐dependent cytocompatibility in HFF cells were systematically evaluated. By demonstrating the successful co‐incorporation of *Ginkgo biloba* extract and CDOTs while maintaining their formulation‐level release and optical characteristics, this study provides a proof of concept for a fluorescent dual‐loaded nanocarrier. Because brain delivery and disease‐specific biological activity were not directly investigated, these potential applications require confirmation through dedicated blood–brain barrier, neuronal, and in vivo studies. Subsequent studies should therefore evaluate the formulation in disease‐relevant neuronal and neuron–glia models before progression to in vivo efficacy studies.

## 2 Materials and Methods

### 2.1 Materials

The raw material in this research is Citric Acid Monohydrate C_6_H_8_O_7_ and urea CH_4_N_2_O, which were purchased from Merck (Germany). Phosphate‐buffered saline (PBS), chloroform, dimethyl sulfoxide (DMSO), Span 60, cholesterol, sodium dodecyl sulfate (SDS), and methanol were purchased from Millipore Sigma (Burlington, MA, USA) and used without further purification. A dialysis membrane (MWCO 12,000 Da) was acquired from Merck Chemical Co. (Darmstadt, Germany). The HFF cell line was purchased from the Pasteur Institute Cell Bank, Tehran, Iran.

### 2.2 Synthesis of CDOTs

The primary substances utilized in this study were citric acid and urea. Citric acid served as a carbon source, whereas urea functioned as a nitrogen source. Citric acid and urea were combined in varying ratios and subsequently dissolved in 60 mL of distilled water, as outlined in Table 1. The process of homogenization involved the application of a hotplate stirrer set at a temperature of 70°C, operating at 600 rpm for a duration of 15 min. This was subsequently complemented by an ultrasonic bath maintained at 40°C for 20 min. The uniformly mixed solution was placed in a microwave reactor. The apparatus employed for the synthesis in this investigation was a Samsung MS28J5255UB apparatus. The formulation was prepared at a reactor power of 600 watts for a duration of 10–13 min.

**TABLE 1:** Synthesis of different citric acid:urea ratio and investigation of quantum yield (%).

Run	Citric acid:urea ratio	Quantum yield (%)
1	1:0	24.11
2	1:1	39.08
3	1:2	47.32
4	1:3	54.75

### 2.3 Calculation of the Quantum Yield (QY)

The quantum yield (QY) represents the ratio of emitted photons to absorbed photons generated by the fluorescent material. The quantum yield of CDOTs can be assessed by utilizing quinine sulfate as a reference fluorescence standard for comparison. The CDOTs were solubilized in purified water, whereas quinine sulfate was solubilized in 0.1 M sulfuric acid solution (quantum yield = 0.54; refractive index = 1.33) [23]. The calculation of QY is derived from the subsequent equation:

(1) $$QY_{c}=QY_{c}\times \frac{I_{c}}{I_{s}}\times \frac{A_{s}}{A_{c}}\times {\left(\frac{\eta_{c}}{\eta_{s}}\right)}^{2}$$

In this context, QY denotes the quantum yield, *I* the integrative fluorescence area, *A* the absorbance intensity at the excitation wavelength of 405 nm, and *η* the refractive index of the solvent. The subscript ‘*c*’ denotes CDOTs, while ‘*s*’ signifies the quinine sulfate substance. The absorbance intensity (*A*) was determined through analysis conducted using a UV–Vis spectrophotometer. The integrative fluorescence area (*I*) was derived through experimentation using an OMA spectrophotometer and subsequently analyzed using the Origin software.

### 2.4 Optimization of Niosomal Formulations Using Response Surface Methodology

Optimizing niosomal formulations was conducted using response surface methodology through a Box–Behnken design. To explore the correlation between a collection of independent factors and those that are dependent, a polynomial equation was employed for fitting the data. Three numerical factors were chosen for analysis: the ratio of amphiphilic molecules to CDOT and Ginkgo, the ratio of surfactant to cholesterol, and the duration of sonication in minutes. The focus was on assessing how variations in these concentrations influenced niosomal particle size in nanometers, the polydispersity index, the entrapment efficacy percentage, and the release percentages of CDOT and Ginkgo. The polynomial equation was derived utilizing Design‐Expert software (Version 13, Stat‐Ease Inc., Minneapolis, MN, USA). Comparative analyses were conducted between the empirical data and the anticipated outcomes. The optimum formulation was selected for further investigation utilizing the point prediction method. Table 2 illustrates these variables along with their respective levels.

**TABLE 2:** Design of experiments using the Box–Behnken method to optimize the niosomal formulation.

Std	Run	Factor 1	Factor 2	Factor 3	Response 1	Response 2	Response 3	Response 4	Response 5	Response 6
		A: Amphiphilic molecules:CDOT&Ginkgo	B: Surfactant:Cholesterol	C: Sonication time	Size	PDI	EE (CDOT)	EE (Ginkgo)	Release (CDOT)	Release (Ginkgo)
		(molar ratio)	(molar ratio)	(min)	(nm)		(%)	(%)	(%)	(%)
8	1	1	0	1	272	0.3	89	91.7	95.3	90.4
6	2	1	0	−1	385	0.33	94.5	95.6	82.5	86.3
10	3	0	1	−1	286	0.3	87.8	86.4	75.4	73.5
5	4	−1	0	−1	341	0.4	88.6	93.4	72	70.6
3	5	−1	1	0	262	0.2	87.3	89.1	83.2	75.5
2	6	1	−1	0	367	0.39	86.2	90	73.5	77.7
12	7	0	1	1	244	0.27	84.4	84.5	78.6	87.5
9	8	0	−1	−1	332	0.38	85.5	84.7	71.4	66.5
11	9	0	−1	1	279	0.32	86.1	82.3	87.3	77.3
15	10	0	0	0	240	0.23	90.3	95.2	85.6	83.5
1	11	−1	−1	0	295	0.28	83.2	84.7	70.2	78.5
4	12	1	1	0	303	0.32	95.2	97.5	93.4	94.5
13	13	0	0	0	246	0.23	93.3	98.7	94.2	88.2
7	14	−1	0	1	231	0.19	87.7	88.3	74.5	79.8
14	15	0	0	0	262	0.22	91.6	96.4	91.8	86.6

We chose the non‐ionic surfactant along with cholesterol using a combined approach comprising preliminary screening and optimization studies and literature review. Using surfactants with low hydrophilic–lipophilic balance (HLB) values, the literature review revealed Span 60 as an ideal niosomal vesicle‐forming surfactant because it maintains high phase transition temperatures and low membrane permeability [24]. The presence of cholesterol strengthens and stabilizes the bilayer membrane, which leads to decreased drug leakage and enhanced drug retention [25]. Preliminary trials with various surfactant combinations were conducted to identify the optimal parameters for niosomal structure development; the results showed that Span 60 with cholesterol provided the best results. Therefore, it was aimed to improve the *Ginkgo biloba* extract and carbon dots dual encapsulation formulation by analyzing response surfaces using response surface methods.

### 2.5 Preparation of Niosomal Formulations

CDOTs@Ginkgo@Niosomes were synthesized using the thin‐film hydration technique [26]. In summary, specified quantities of CDOTs, cholesterol, and Span60 were solubilized in 9 mL of a chloroform/methanol mixture at a ratio of 2:1 (v/v). The mixture was carefully transferred to a 50 mL round‐bottom flask. The organic solvent was evaporated under vacuum conditions using a rotary evaporator (Heidolph Instruments, Schwabach, Germany) set at 60°C and a speed of 120 rpm for 60 min, resulting in the formation of a thin dried film at the bottom of the flask. The thin film was rehydrated in a phosphate‐buffered saline solution infused with 10 mg Ginkgo, maintained at 60°C for 60 min, accompanied by gentle agitation at 120 rpm. To achieve the optimum size distribution for CDOTs@Ginkgo@Niosomes, niosomal suspensions underwent sonication using a probe sonicator (UP50H compact laboratory homogenizer, Hielscher Ultrasonics, Teltow, Germany) for 7 min. The samples were maintained at 4°C for the analysis of various niosomal formulations.

### 2.6 Physical Characterization

The dimensions of the particles, zeta potential, and polydispersity index of the niosomal formulations were assessed at 25°C utilizing a benchtop dynamic light scattering apparatus (operating at 25°C and a wavelength of 633 nm) (Zetasizer Nano S90, Malvern Panalytical Ltd., Malvern, United Kingdom). The AFM analysis was conducted in contact mode using a NanoScope E system manufactured by Digital Instruments (USA). The surface morphology was analyzed using scanning electron microscopy (SEM) employing a Topcon instrument. The samples were subjected to gold coating with a thickness of 16 nm utilizing a Bal‐tec system from the USA.

### 2.7 Chemical Characterization

The analysis of the absorption spectrum was conducted utilizing a UV–Vis spectrophotometer (Alpha Series Bruker, USA). The emission spectra were analyzed using optical multi‐analysis (OMA), employing a laser with a wavelength of 405 nm as the excitation source. Fourier‐transform infrared spectroscopy (FTIR) (Spectrum Two, USA) was employed to investigate the molecular interactions among CDOTs, Ginkgo, and niosomes. For this experiment, lyophilized specimens were individually combined with KBr, and the resulting pellets were created by applying pressure using a hydraulic press. FTIR analyses were conducted within the scanning range of 4000–400 cm^−1^, maintaining a constant resolution of 4 cm^−1^, and performed at room temperature. DSC analysis was performed within the temperature range of 25°C to 300°C at a heating rate of 10 K/min under a nitrogen flow of 50 mL/min utilizing a METTLER TOLEDO instrument.

### 2.8 Entrapment Efficacy

The niosomal formulations underwent ultrafiltration using an Eppendorf 580R centrifuge from Hamburg, Germany, using an Amicon Ultra−15 membrane, operating at 4000 rpm for 30 min at 4°C. The non‐entrapped CDOTs and Ginkgo were isolated from the entrapped CDOTs and Ginkgo to quantify the entrapment efficacy percentage (EE%). The concentrations of CDOTs and Ginkgo were assessed through the measurement of absorption at wavelengths of 200 and 277 nm, respectively, utilizing a UV–visible light spectrophotometer (JASCO, V‐530, Tokyo, Japan). The subsequent equation was employed to determine the EE%:

(2) $$\mathrm{EE}\%=\left(\frac{A-B}{A}\right)\times 100$$

where *A*, initial drug concentration for niosomal preparation; *B*, the concentration of non‐entrapped drugs after centrifugation.

### 2.9 In Vitro Drug Release and Kinetic Study

For the assessment of substance release, 2 mL of each sample was introduced into a semipermeable acetate cellulose dialysis bag with a molecular weight cutoff of 12 kDa. The final sample was subjected to immersion in 50 mL of PBS‐SDS (0.5% w/v; release medium). The mixture was stirred at a rate of 50 rpm using a magnetic stirrer and maintained at a temperature of 37°C for 72 h. At a designated interval, 1 mL of the release medium was extracted and replaced with an equivalent volume of fresh PBSSDS [26]. The quantity of release at specified intervals was quantified using an ultraviolet light spectrophotometer. Various mathematical models were employed to assess the release kinetics of CDOTs and Ginkgo from the samples: the Korsmeyer‐Peppa model (log cumulative % release vs. log time), the Higuchi model (cumulative % release vs. the square root of time), the first‐order model (cumulative % remaining vs. time), and the zero‐order model (cumulative % release vs. time). The values of the correlation coefficient (*r*) acquired through the regression of the plots derived from the earlier‐mentioned models were utilized to compute the linear curve.

### 2.10 Cell Viability

The HFF cell line was obtained from the Pasteur Institute Cell Bank/National Cell Bank of Iran (Tehran, Iran). Cells were cultured in DMEM supplemented with 10% fetal bovine serum (FBS) and 1% penicillin/streptomycin and maintained at 37°C in a humidified atmosphere containing 5% CO_2_. Cultured HFF cells underwent treatment and incubation with varying concentrations of free niosomes, CDOTs, Ginkgo, and CDOTs@Ginkgo@Niosomes (0.1, 0.5, 1, 5, 10, and 50 μg/mL) for a duration of 24 and 48 h. Subsequently, the HFF cells underwent a 4‐h incubation with MTT (0.5 mg/mL) to facilitate the breakdown of the colorless tetrazolium dye MTT into insoluble formazan. The absorbance of the formazan product was measured at a wavelength of 570 nm. To assess the colorimetric properties of the treated cells, formazan was solubilized in 100 μL of DMSO [59]. The following equation was used for quantification:

(3) $$\text{Cell viability}\;\left(\%\right)=\frac{\text{Optical density of treated cells}}{\text{Optical density of control cells}}\times 100\%$$

### 2.11 Scratch Assay

A scratch assay was conducted to examine the influence of Ginkgo and CDOT@Ginkgo @Niosome formulations on cellular migration dynamics. The experiments were performed on a single layer of human fibroblast cells. The cells were introduced into a 6‐well plate at a density of 2 × 10^5^ cells per well and allowed to proliferate until confluence was achieved. A scratch was introduced in the monolayer using a sterile pipette tip at *T* = 0, followed by washing the wells with PBS to eliminate any dislodged cells. The cells were subsequently subjected to incubation in DMEM enriched with 10% FBS and 1% antibiotics (penicillin/streptomycin) at a temperature of 37°C and a CO_2_ concentration of 5%. Photographs of the scratch were obtained at *T* = 0, *T* = 24, and *T* = 48 h using an inverted phase‐contrast microscope.

### 2.12 Statistical Analysis

Data are presented as mean ± SD from at least three independent experiments. Statistical analysis was performed using GraphPad Prism software. Comparisons among multiple groups were performed using one‐way ANOVA, followed by Tukey's post hoc test. A *p*‐value < 0.05 was considered statistically significant.

## 3 Result and Discussion

### 3.1 Synthesis of CDOTs as Fluorescence Material

The primary attribute of CDOTs is their fluorescence, which serves as the standard for evaluating the efficacy of the synthesis process. A preliminary investigation was carried out to analyze the fluorescence effects of CDOTs C1‐4 (Table 1). The main compounds employed in this investigation were citric acid and urea. Citric acid acts as a source of carbon, while urea provides nitrogen. The combination of citric acid and urea was executed in different ratios (Figure 1a). The mixture was synthesized in a reactor at a power of 600 watts over a period of 10 to 13 min.

**FIGURE 1:**
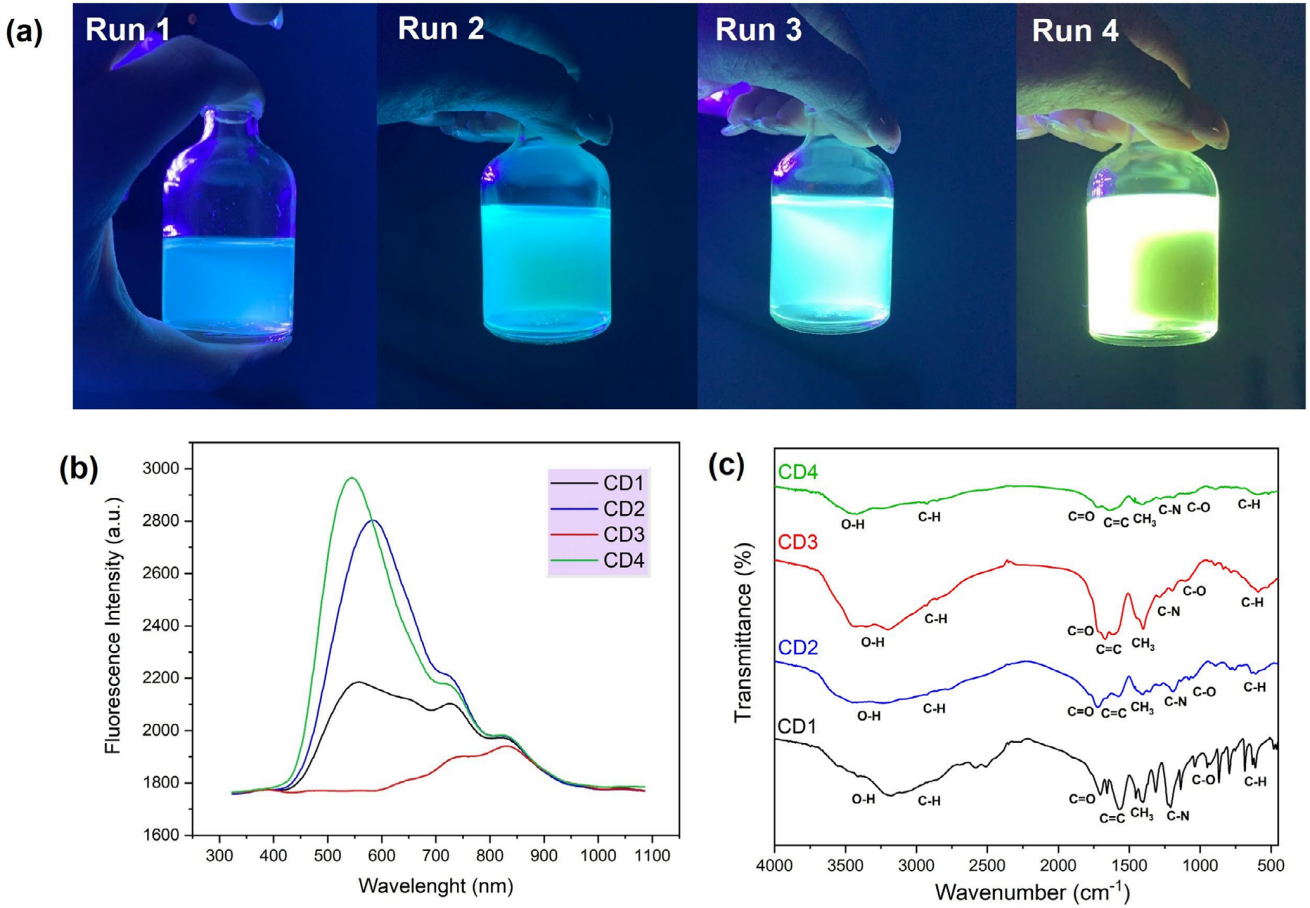
Photograph images of the prepared CDOT with different citric acid:urea ratio (Run 1: 1:0, Run 2: 1:1, Run 3: 1:2, Run 4: 1:3) under UV light (a), Evolution of the fluorescence spectra of the prepared CDOT with different citric acid:urea ratio (b), and FTIR spectra of the prepared CDOT with different citric acid:urea ratio (CD1: 1:0, CD2: 1:1, CD3: 1:2, CD4: 1:3) (c).

Figure 1b illustrates the fluorescence intensity spectra of four distinct carbon dot samples (CD1 to CD4), each produced using different ratios of citric acid to urea. The horizontal axis represents the wavelength (nm) of the emitted light, whereas the vertical axis reflects the fluorescence intensity (a.u.). The lowest fluorescence intensity, corresponding to a quantum yield of 24.11%, was observed for CD1, which was prepared at a citric acid:urea ratio of 1:0 and is therefore consistent with the absence of urea‐derived nitrogen doping. The fluorescence intensity exhibits a notable enhancement with a quantum yield of 39.08% when CD2 is mixed in a 1:1 ratio. The incorporation of nitrogen via urea significantly enhances the quantum yield. The introduction of CD3 at a 1:2 ratio results in a quantum yield of 47.32%, significantly enhancing the fluorescence intensity. The enhancement of nitrogen incorporation is evident, and the optical properties are remarkable. The peak fluorescence intensity is exhibited by the CD4 at a 1:3 ratio, with a quantum yield of 54.75%. The data indicate that an increased concentration of urea improves fluorescence performance and promotes efficient nitrogen doping.

The FTIR spectrum of CDOTs is shown in Figure 1c. According to the spectrum assessment, the CDOTs samples exhibited O—H bonds at 3300 and 3150 cm^−1^, indicating the presence of numerous hydroxyl groups on the surface of the CDOTs [15]. Analyzing the surface characteristics of CDOTs reveals the presence of C—H bonds at frequencies of 2990 cm^−1^, 1403 cm^−1^, and 700–800 cm^−1^, alongside C=O bonds observed at 1715 cm^−1^, indicating the existence of a carboxyl group [16]. The CO functional group on the surface of CDOTs was observed at an absorption of 1058 cm^−1^, while the CN functional group was detected at 1188 cm^−1^, and the C=C functional group was noted at 1575 cm^−1^. The identification of diverse functional groups on the surface of CDOTs through FTIR reveals a range of energy levels, leading to the formation of multiple emission traps. The incorporation of nitrogen onto the surface of CDOTs introduces additional energy levels that act as emissive traps. The revised energy state exhibits a reduced interval between the highest occupied molecular orbital (HOMO) and lowest unoccupied molecular orbital (LUMO). Upon excitation of CDOTs with equivalent energy, a greater number of electrons are elevated, resulting in an increased emission intensity [21].

### 3.2 Formulation of CDOT@Ginkgo@Niosomes

In this experiment, the molar ratio of amphiphilic molecules to CDOT and Ginkgo (A), the molar ratio of surfactant to cholesterol (B), and the duration of sonication (C) were selected as the independent variables. To achieve optimization, the following parameters were selected: nanoparticle size, polydispersity index, encapsulation efficiency percentage, and release percentage. According to Table 2, the particle size of CDOT@Ginkgo@Niosomes was measured to be between 231 and 385 nm. Figure 2a illustrates the response surface plot depicting the particle size of CDOT@Ginkgo@Niosomes. Elevating the molar ratio of amphiphilic molecules relative to CDOT and Ginkgo results in an augmentation of particle size. Elevating the molar ratio of surfactant to cholesterol, along with extending the sonication duration, results in a reduction of particle size. As illustrated in Table 2, the polydispersity index of CDOT@Ginkgo@Niosomes ranges from 0.19 to 0.4. Figure 2b illustrates the PDI response surface plot for CDOT@Ginkgo@Niosomes. As the molar ratio of amphiphilic molecules relative to CDOT and Ginkgo is elevated, there is a corresponding increase in the polydispersity index (PDI). Elevating the molar ratio of surfactant to cholesterol, along with extending the sonication duration, results in a reduction of the polydispersity index (PDI). The data presented in Table 2 indicate that the encapsulation efficiency (EE %) of CDOT falls within the range of 83.2% to 95.2%. The response surface plot of EE (%) for CDOT is depicted in Figure 2c. Increasing the molar ratio of amphiphilic molecules relative to CDOT and Ginkgo results in a rise in the encapsulation efficiency (EE %) of CDOT. With an increase in the molar ratio of surfactant to cholesterol and an extended sonication time, the encapsulation efficiency (EE %) of CDOT initially rises before subsequently declining. The data presented in Table 2 indicate that the extraction efficiency of Ginkgo falls within the range of 82.3% to 98.7%. The response surface plot of extraction efficiency (EE %) for Ginkgo is depicted in Figure 2d. Increasing the molar ratio of amphiphilic molecules relative to CDOT and ginkgo enhances the encapsulation efficiency (EE %) of ginkgo. With an increase in the molar ratio of surfactant to cholesterol and an extension of sonication time, the encapsulation efficiency (EE %) of Ginkgo initially rises before subsequently declining. The data presented in Table 2 indicate that the release percentage of CDOT from nanoparticles falls between 70.2% and 95.3%. According to Figure 2e, the percentage release of CDOT from nanoparticles exhibited an increase with the rising molar ratio of amphiphilic molecules relative to CDOT and Ginkgo. With an increase in the molar ratio of surfactant to cholesterol and an extension of sonication time, the release percentage of CDOT from nanoparticles initially rises before subsequently declining. The percentage of Ginkgo released from nanoparticles ranges from 70.6% to 94.5%, as illustrated in (Table 2). As illustrated in Figure 2f, a rise in the molar ratio of amphiphilic molecules relative to CDOT and Ginkgo resulted in an enhanced release percentage of Ginkgo from the nanoparticles. With an increase in the molar ratio of surfactant to cholesterol and an extension of sonication time, the release percentage of Ginkgo from nanoparticles initially rises before subsequently declining. According to the desirability criteria, the optimized formulation was successfully prepared and characterized. Thus, the optimized formulation was used for further experiments. The DLS‐derived particle size distributions and zeta potential measurements of the optimized niosomal formulations are presented in Supporting Information Results, Section S1 (Figure S1).

**FIGURE 2:**
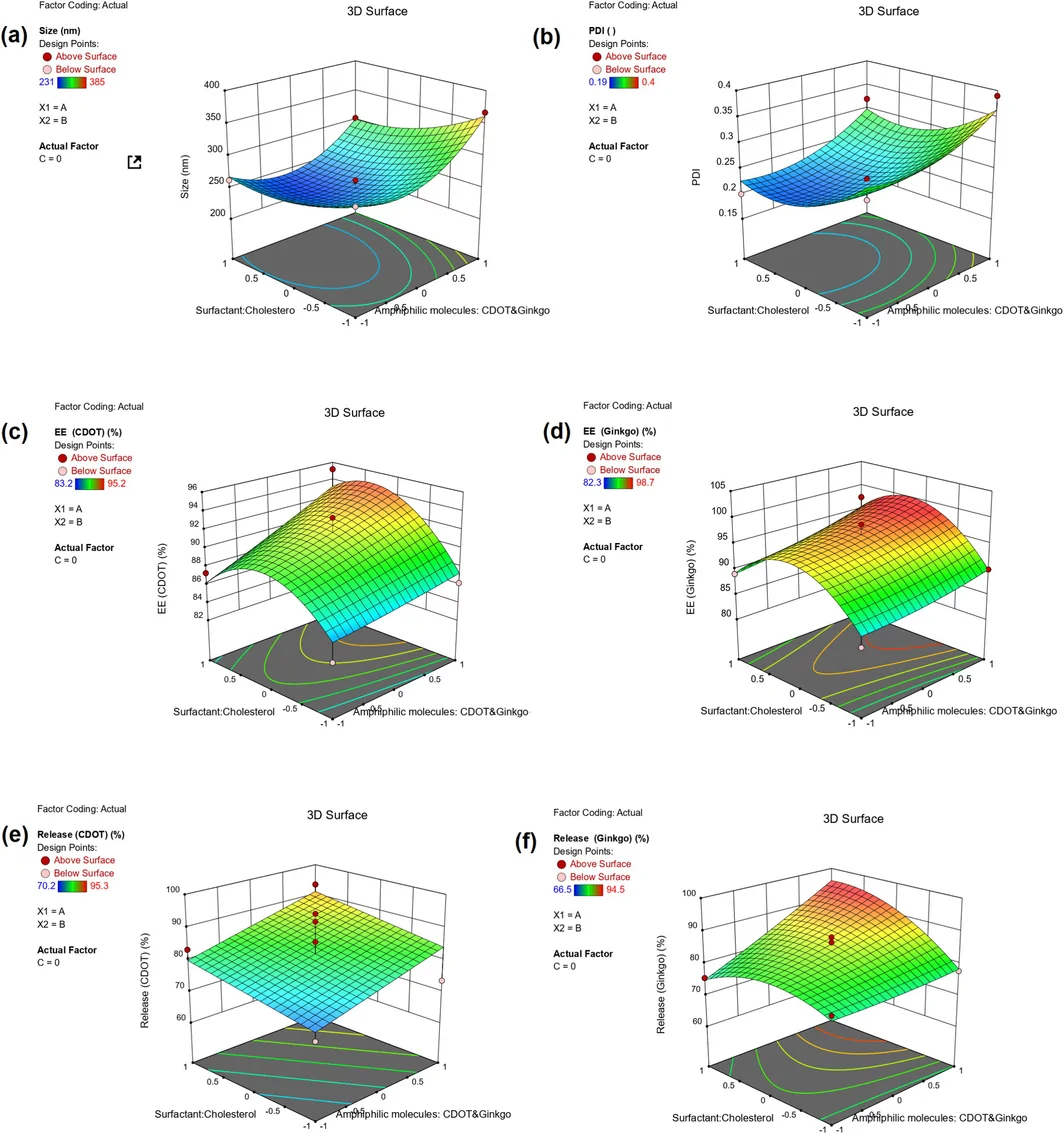
Response surfaces illustrating the relationship between the molar ratio of amphiphilic molecules to CDOT@Ginkgo and the molar ratio of surfactant to cholesterol: (a) size, (b) PDI, (c) EE of CDOT (%), (d) EE of Ginkgo (%), (e) release of CDOT (%), and (f) release of Ginkgo (%).

### 3.3 Size Distribution and Morphological Studies of Optimized Niosomes

The morphology of the synthesized niosomes was examined using FESEM. The niosomes and CDOTs@Ginkgo@Niosomes formulations exhibited a spherical, smooth, and uniform morphology, as observed in the FESEM images (Figure 3a,b). The mean diameters of the niosomes and CDOTs@Ginkgo@Niosomes formulations were 69.56 and 72.19 nm, respectively (Figure 3c,d). The particle size of CDOTs@Ginkgo@Niosomes was greater than that of bare niosomes. Ginkgo could be incorporated within the bilayer, influencing its interaction with the bilayer. The dimensions of the niosomes observed through SEM were less than those quantified by the Nano Zetasizer. This difference between electron microscopy‐derived diameters and DLS‐based hydrodynamic diameters may be attributed to sample drying during electron microscopy preparation, the hydration shell surrounding vesicles in aqueous dispersion, possible vesicle swelling in the hydrated state, and the intensity‐weighted nature of DLS measurements. The AFM images depicting the niosomes and CDOTs@Ginkgo@Niosomes formulation are presented in Figure 3e–j. The niosomes exhibit a spherical morphology; however, a minor change in vesicle size was observed, possibly owing to the fusion of niosomes due to interactions with the mica substrate and the surfaces of the niosomes. The sizes of the niosomes exhibited no notable variation across the various methods employed. The free niosome (Figure 3g) has a lower *R*_*q*_ value, showing minimal surface roughness, while the CDOTs@Ginkgo@Niosomes (Figure 3j) have a higher *R*_*q*_, indicating increased surface roughness due to the incorporation of the CDOTs and Ginkgo extract into the niosomal structure.

**FIGURE 3:**
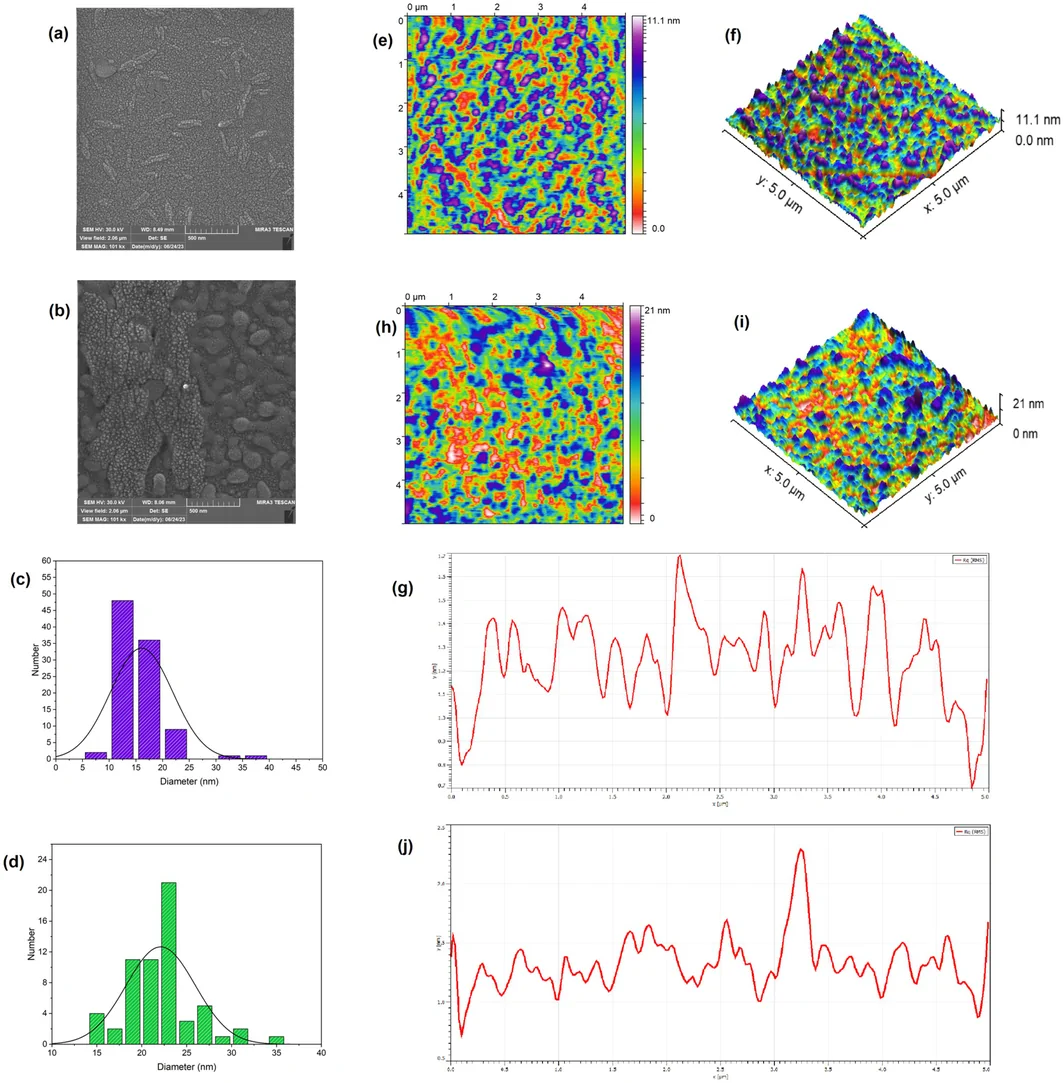
FESEM images of free noisome and CDOTs@Ginkgo@Niosomes (a, b), size distribution of free noisome and CDOTs@Ginkgo@Niosomes using ImageJ software (c, d), 2D, 3D, and the *R*_*q*_ measurement of AFM images of free noisome (e–g) and CDOTs@Ginkgo@Niosomes (h–j).

### 3.4 Structural Analysis of Optimum Niosomal Formulation

Figure 4a shows the main characteristic peaks for FTIR spectra of different samples. Cholesterol exhibits absorption bands at 1643 cm^−1^ (C=C stretch), 2928 cm^−1^ (C—H stretch), 3407 cm^−1^ (O—H stretch), 1024–1364 cm^−1^ (CH_2_ bending and CH_2_ deformation), 1462 cm^−1^ (C—C stretching in the aromatic ring), and 1646 cm^−1^ (C=C stretch). Span 60 shows peaks at 1171 cm^−1^ (C—O stretch), 2838–2922 cm^−1^ (C—H stretch), and 3420 cm^−1^ (O—H stretch). For Ginkgo, the peaks at 1029, 1630, 2928, and 3380 cm^−1^ can be attributed to the C—O stretch, C=C stretch, C—H stretch, and O—H stretch, respectively. Niosome shows peaks at 1080 cm^−1^ (C—O stretch), 1024–1350 cm^−1^ (CH_2_ bending and CH_2_ deformation), 1636 cm^−1^ (C=O stretch), 1753 cm^−1^ (C=C stretch), 2832–2950 cm^−1^ (C—H stretch), and 3445 cm^−1^ (O—H stretch). Ginkgo@Niosome shows absorption bands at 1040–1390 cm^−1^ (CH_2_ bending and CH_2_ deformation), 1740 cm^−1^ (C=C stretch), 2826–2950 cm^−1^ (C—H stretch), and 3435 cm^−1^ (O—H stretch). CDOT@Ginkgo@Niosome combines these features with distinctive peaks for CDOT (1636 cm^−1^ for C=O, 1022 cm^−1^ for C—O, and 1404 cm^−1^ for C—O—H) alongside Ginkgo and niosome. Figure 4b shows the DSC of CDOT@Ginkgo@Niosome, which shows a notable endothermic peak at approximately 46.51°C. This may correspond to the melting of the noisome bilayers or the phase transition of the encapsulated compounds (such as CDOT and Ginkgo extract) within the noisome structure. The transition onset starts at around 35.05°C and ends at 73.36°C, indicating a broader thermal transition range. Cholesterol typically stabilizes the niosomal structure by affecting the phase transition temperature of the lipid bilayers. Figure 4c shows a physical image of the CDOT@Ginkgo@Niosome sample. The niosomal formulation in the vial appears to exhibit some level of luminescence, which is likely due to the presence of CDOTs. The luminescence observed in the CDOT@Ginkgo@Niosome vial suggests that the optical properties of CDOTs were at least partially preserved after incorporation into the niosomal formulation. This observation is consistent with the presence of fluorescent CDOTs in the formulation. However, this result should be interpreted only as formulation‐level fluorescence evidence. It does not demonstrate cellular uptake, real‐time biological tracking, biodistribution, or diagnostic imaging capability. The incorporated CDOTs preserved detectable fluorescence in the formulation, suggesting that the system may be useful for future imaging/tracking studies after appropriate biological validation.

**FIGURE 4:**
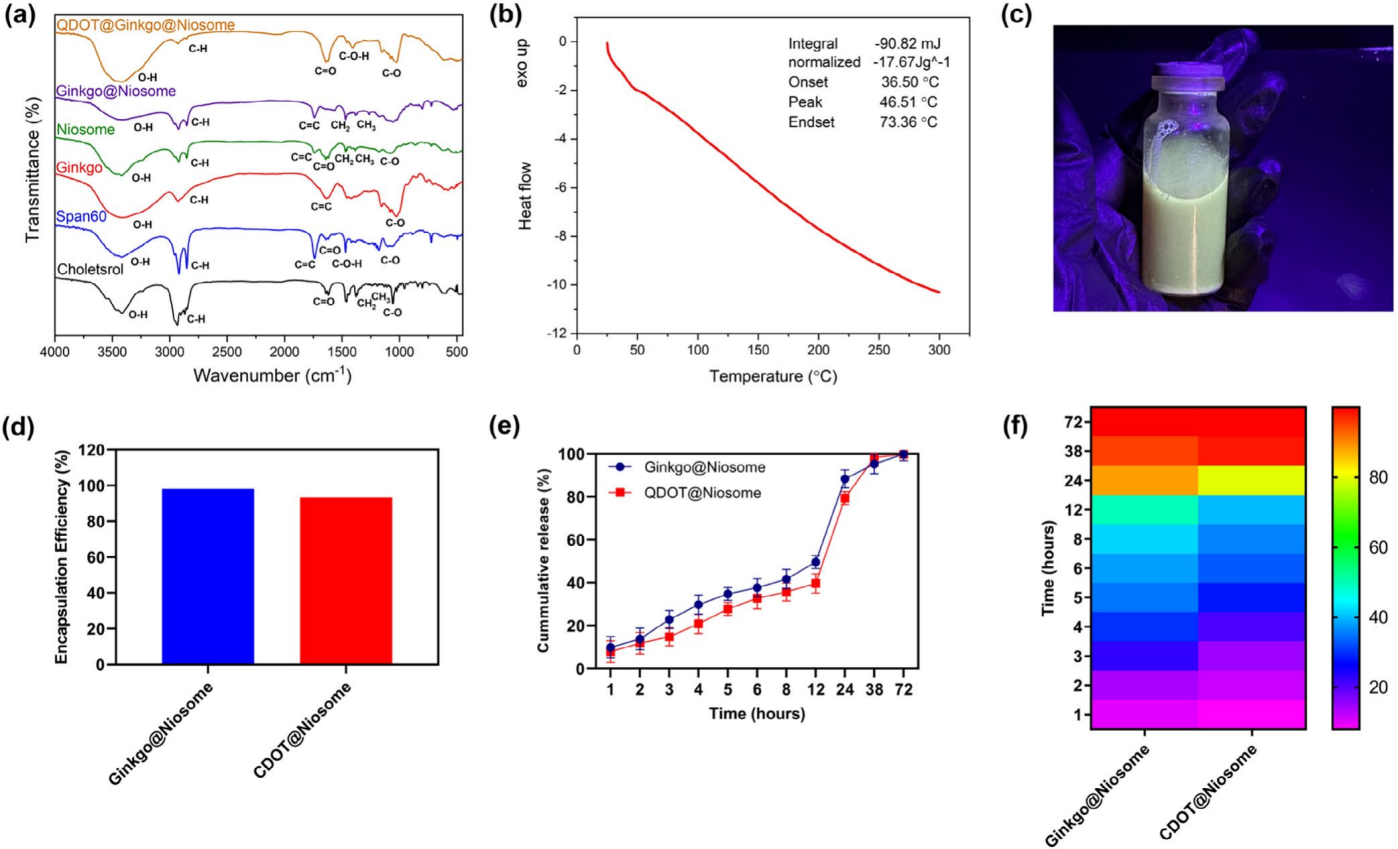
FTIR spectra of Cholesterol, Span 60, Ginkgo, Niosome, Ginkgo@Niosome, and CDOT@Ginkgo@Niosome (a), DSC image of CDOT@Ginkgo@Niosome (b), photograph image of the prepared C CDOT@Ginkgo@Niosome (c), encapsulation efficiency (%) of Ginkgo@Niosome and CDOT@Niosome (d), release profile of CDOT and Ginkgo from niosome (e, f).

### 3.5 Investigation of Release Profile of Niosomal Formulation

As shown in Figure 4d, the encapsulation efficiency of Ginkgo@Niosome and CDOT@Niosome was 98.14 and 93.22, respectively. The release profile investigation was conducted to analyze the influence of niosomes on the release kinetics of CDOTs and Ginkgo from the vesicular structures. The attainment of an optimal CDOTs and Ginkgo release system constituted another objective of the study, achieved through the application of release kinetic models. The corresponding release kinetic model fits, including zero‐order, first‐order, Higuchi, and Korsmeyer–Peppas models, are presented in Supporting Information Results, Section S2 (Figure S2).

Figure 4e,f shows the cumulative release profiles of CDOTs and Ginkgo from the optimized niosomal formulations under physiological pH conditions. The tested formulations were Ginkgo@Niosome and CDOT@Niosome, respectively. Released CDOTs were quantified using fluorescence spectroscopy, whereas Ginkgo extract was quantified as Ginkgo extract equivalents. Blank niosomes and single‐component controls were used for the background correction.

Figure 4e,f illustrates the quantities of CDOTs and Ginkgo preserved within the various niosome formulations across designated time intervals. The findings demonstrated that CDOTs and Ginkgo were released from the niosomal formulations in a delayed manner over 72 h, with near‐complete release observed by the final time point. The release quantities from all niosomal formulations were measured at approximately 49.65% and 39.72% over the initial 12 h for Ginkgo@Niosome and CDOT@Niosome, respectively. This indicates that Ginkgo@Niosome exhibits a marginally elevated release compared to CDOT@Niosome over a 12‐h period, and it also demonstrates a greater overall release. Subsequent to this duration. The release kinetics exhibited an enhancement, with observations indicating 99.91% and 99.82% release rates across all formulations after a 72‐h period. Sahrayi et al. also observed that Letrozole and Cyclophosphamide from the solutions exhibit a rapid release in the PBS medium during the initial hour as these compounds go through the diffusion gradient. Nevertheless, the drug‐loaded niosomal formulations inhibited this occurrence, resulting in a sustained release of Letrozole and Cyclophosphamide [27].

### 3.6 Cytotoxicity Study

The formulations for the cytotoxicity study, featuring varying concentrations (0.1–50 μg/mL), were illustrated in Figure 5, specifically targeting HFF cell lines. The results indicate that all formulations exhibit no toxicity at low concentrations after a duration of 48 h (0.1, 0.5, 1, and 5 μg/mL). At concentrations of 10 and 50 μg/mL, CDOT@Ginkgo@Niosome exhibits a modest cytotoxic effect on HFF cell lines, with statistical significance noted (*p* < 0.05 at 10 μg/mL and *p* < 0.0001 at 50 μg/mL). These data indicate preliminary concentration‐dependent cytocompatibility in HFF cells. Although the niosomal formulation showed acceptable cytocompatibility at lower concentrations, the observed reduction in cell viability at higher concentrations indicates that the formulation cannot be considered broadly biocompatible without further evaluation in additional cell models, hemocompatibility assays, inflammatory‐response assays, and in vivo safety studies [26]. In a study, Sahrayi et al. demonstrated that the unloaded niosomes exhibited biocompatibility at the concentrations evaluated, with no notable impact on MCF10A cell viability identified after a 72‐h period [27]. In another study, the cytotoxicity of empty niosomes, tetracycline hydrochloride‐niosomes, and tetracycline hydrochloride‐niosomes @chitosan was assessed against HFF cells utilizing the MTT assay. The empty niosomes exhibited negligible toxicity towards the HFF cell line. They also confirmed that the absence of toxicity in free niosomes renders them exceptionally attractive for use as nanocarriers. The investigation revealed that the viability of HFF cells remained unaffected when subjected to tetracycline hydrochloride‐niosome or tetracycline hydrochloride‐niosome@chitosan at higher concentrations, even following a treatment duration of 48 h [28]. Because HFF fibroblasts are not neuronal, glial, or BBB‐forming cells, these results should be interpreted only as preliminary cytocompatibility data. They do not establish neuroprotective activity, neuronal safety, BBB compatibility, or Alzheimer's disease‐relevant therapeutic efficacy, and these features should be assessed in future studies.

**FIGURE 5:**
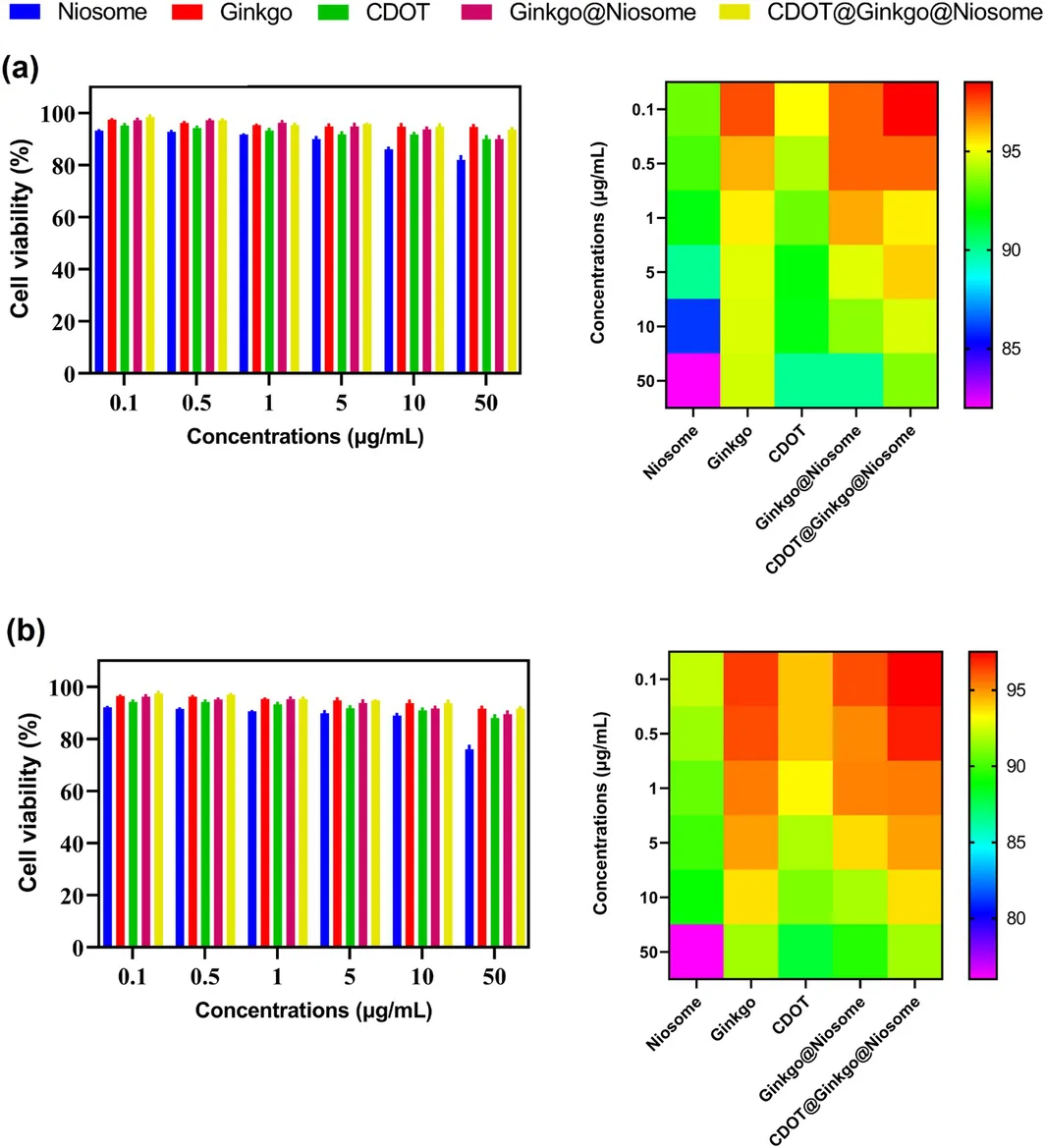
MTT Assay of Niosome, Ginkgo, CDOT, Ginkgo@Niosome, and CDOT@Niosome formulations on HFF cell lines after 24 (a) and 48 h (b).

#### 3.6.1 Preliminary Fibroblast Migration Assay

The findings indicated that both Ginkgo and CDOT@Ginkgo@Niosome exhibited a notable influence on cell migration (Figure 6). At *T* = 0, the scratch width exhibited uniformity across all wells, suggesting an even distribution of cells within the monolayer. After a period of 48 h, the measurement of scratch width in the wells treated with Ginkgo and CDOT@Ginkgo@Niosome exhibited a notable reduction compared to the control wells. This indicates that the Ginkgo and CDOT@Ginkgo@Niosome facilitated cellular migration and proliferation, resulting in accelerated wound closure. Overall, these findings indicate that Ginkgo and CDOT@Ginkgo@Niosome may serve as promising agents in the domains of wound healing and tissue regeneration, where the enhancement of cell migration and proliferation is essential for effective tissue repair. In a study conducted by Karavelioglu et al., SH‐SY5Y cells were subjected to scratch assays and subsequently treated with chitosan NPs and Ginkgo‐chitosan NPs. Wells were monitored at designated intervals (3, 6, 8, 24 h). A more rapid wound closure occurred in cells exposed to Ginkgo‐chitosan NPs compared to those treated with chitosan NPs and the untreated control group. A Given the close relationship between cell migration and proliferation, this finding further substantiates the influence of the CDOT@Ginkgo@Niosome on cell proliferation [29]. As HFF fibroblasts are not an Alzheimer's disease‐relevant neuronal or glial model, the scratch assay findings should be interpreted only as preliminary evidence of cytocompatibility and fibroblast migratory behavior. The data for demonstration of neuroprotective activity, blood–brain barrier transport, or therapeutic efficacy in Alzheimer's disease can be evaluated in future studies.

**FIGURE 6:**
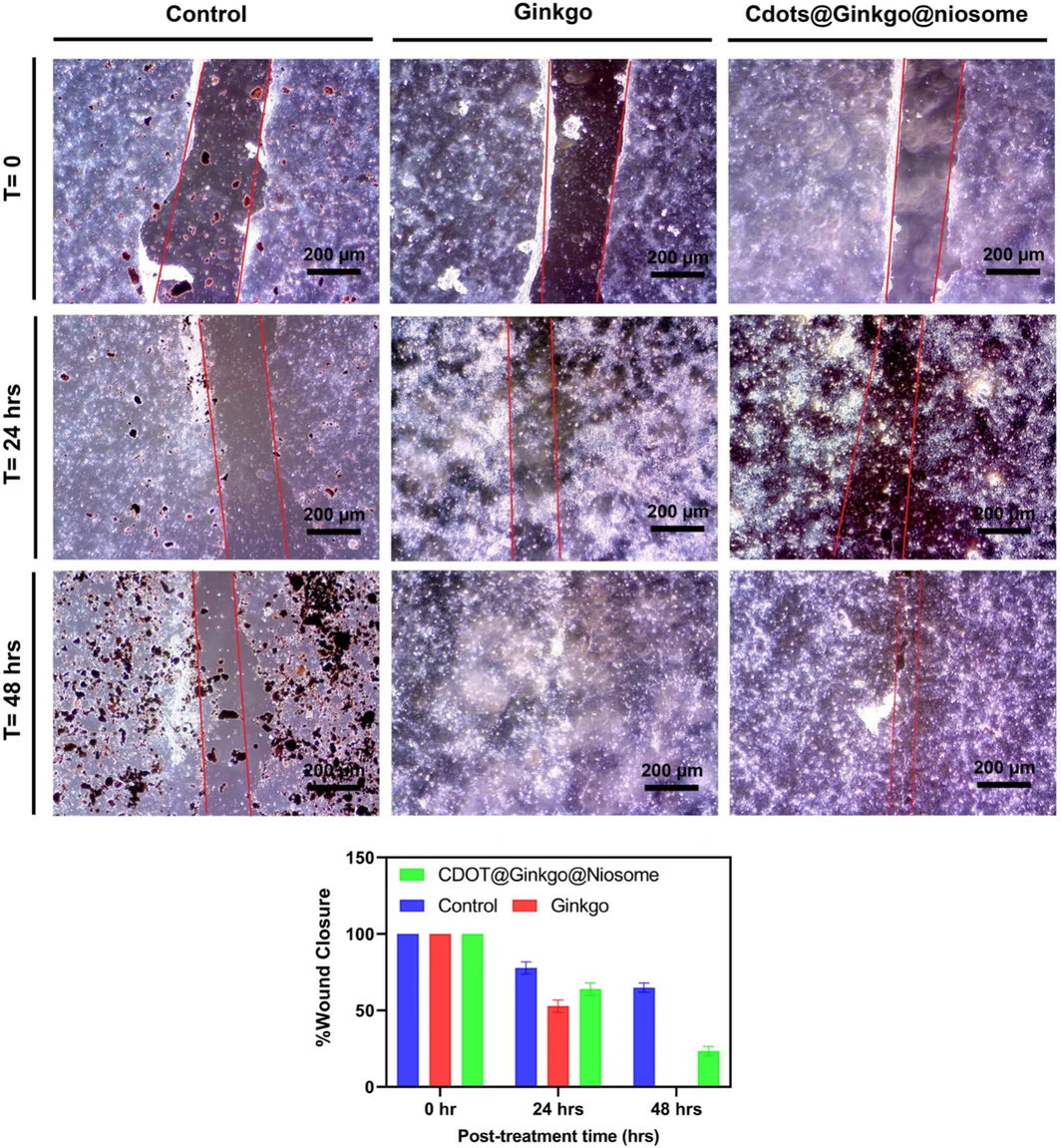
Scratch assay and wound closure (%) at 24 and 48 h after the treatment with Ginkgo and CDOT@Niosome formulations.

## 4 Conclusion

In this study, dual‐loaded CDOT@Ginkgo niosomes were formulated and characterized to obtain a fluorescent natural product formulation, formulation level fluorescence, delayed release nature, and preliminary cytocompatibility. The optimized nanocarriers were characterized based on their morphology, particle size, zeta potential, entrapment efficiency, and release profile. The blank niosomes had a zeta potential of −43.9 ± 0.556 mV, which shifted to −23.96 ± 0.702 mV after loading with CDOTs and Ginkgo, whereas the particle diameter increased from the blank niosomes to 129 nm to 380 nm after loading. Both formulations exhibited high apparent entrapment efficiency, prolonged/relaxed release of Ginkgo (49.65% released in 1, 12 h) and CDOT (39.72% released in 1, 12 h) within 72 h. Preliminary cytocompatibility testing in HFF cells indicated good cytocompatibility at lower concentrations; however, further testing for safety is needed before more extensive biocompatibility claims can be made. Importantly, the present study did not investigate blood–brain barrier transport, neuronal uptake, neuroprotection, amyloid‐β/tau‐related mechanisms, and therapeutic efficacy in an Alzheimer's disease model.

No experiments assessing blood–brain barrier transport, neuronal uptake, neuroprotection, amyloid‐β‐ or tau‐related mechanisms, biological imaging, diagnostic performance, or therapeutic efficacy in an Alzheimer's disease model were conducted. Therefore, the present findings provide a physicochemical and preliminary in vitro foundation for future biological evaluation but do not support claims regarding Alzheimer's disease treatment or prevention, brain delivery, or theranostic performance.

## Author Contributions

Y.M.: conceptualization, methodology, data collection, and manuscript preparation. M.S.S.: supervision, methodology, review, and editing of the manuscript. E.H.: data analysis, software, and visualization. M.E.: investigation, validation, and manuscript review. N.A.: project administration, funding acquisition, and supervision.

## Funding

The authors have nothing to report.

## Ethics Statement

Ethical approval was not required for this study because it did not involve human participants, human identifiable data, or animal experiments. All experiments were conducted in vitro using analytical standards and laboratory procedures. The HFF cell line used in this study was obtained from the Pasteur Institute Cell Bank/National Cell Bank of Iran (Tehran, Iran).

## Consent

The authors have nothing to report.

## Conflicts of Interest

The authors declare no conflicts of interest.

## Supporting information


**Figure S1:** DLS image of free noisome and CDOTs@Ginkgo@Niosomes (a, b) and Zeta potential measurement of free noisome and CDOTs@Ginkgo@Niosomes (c, d).
**Figure S2:** The release kinetics of Ginkgo@Niosome and CDOT@Niosome formulations using Zero‐order, First‐order, Higuchi, and Korsmeyer‐Peppas models.

## Data Availability

All data supporting the findings of this study are available within the paper. If needed, all data is available based on request.
