# Supplementary material for: Development, Optimization, and Physicochemical Characterization of Fluorescent Carbon Dot‐ Ginkgo biloba Extract Co‐Loaded Niosomes for Future Neurological Disease‐Relevant Evaluation
Source: CNS Neurosci Ther. 2026 Nov 1;32(11):e71230. doi: 10.1002/cns.71230 (PMC13725130; doi:10.1002/cns.71230)
Supplement: Supplementary file 1 [file CNS-32-e71230-s001.docx]

**Supplementary Results:**

**Section S1. Particle Size and Zeta Potential of optimized niosomes**

The physicochemical characteristics of vesicular carrier systems, especially their dimensions, morphology, and surface chemistry, are essential in influencing tissue penetration and cell delivery . The morphology of the prepared samples was investigated using techniques such as DLS and zeta potential measurements. The dimensions of the particle prior to and following the drug encapsulation measured 129 nm and 380 nm, respectively (**Figure S1a, b**). As illustrated in **Figure S1b**, there is a noticeable rise in the size of the CDOTs@Ginkgo@Niosomes formulation when compared to the empty noisome. Furthermore, the ζ-potential of niosomes, measured at −43.9 ± 0.556, exhibits a significant alteration upon the incorporation of CDOTs and ginkgo into the niosome formulation, resulting in a new value of −23.96 ± 0.702 (**Figure S1c, d**). The analysis of the data indicates that the formulations achieved an appropriate particle size and maintained a stable dispersion throughout the mixture. The findings were consistent with those observed in other investigations. Rezaei et al. observed a reduction in the particle size of Folic Acid-Decorated pH-responsive Niosomes, noting a reduction from 324.7 nm when the surfactant to cholesterol molar ratio was 6:3 to 174.2 nm at a ratio of 2:1 (Rezaei, Rezaei et al. 2022). All niosome formulations exhibit a negative Zeta Potential, indicating their stability is enhanced by the weak electrostatic repulsive forces present within the niosomal bilayer. The zeta value of empty Niosomes was measured at −27.54 ± 1.34 mV, while the Zeta Potential of Niosomes/5-FU was determined to be −13.45 ± 2.55. In a study, Mansoori-Kermani et al. stated the average sizes of bare niosomes, epirubicin-niosomes, and epirubicin-niosomes-hyaluronic acid groups as 167.2 nm, 186.5 nm, and 225.9 nm, respectively.

Figure S1. DLS image of free noisome and CDOTs@Ginkgo@Niosomes (a,b) and Zeta potential measurement of free noisome and CDOTs@Ginkgo@Niosomes (c,d).

**Section S2.** **Drug release kinetic model of optimum formulation**

During the experimental phase, the release kinetics of the optimal formulation were assessed. Various kinetic models were represented in a linear format derived from the release data. The kinetic model for the optimal formulation was selected due to the regression coefficient being close to 1. The R^2^ values for each model indicated that the release is governed by the mechanisms of diffusion and erosion. According to **Figure S2**, the first-order kinetic model is the most suitable for CDOT, and Ginkgo exhibits kinetic release from the optimal niosomal formulation, likely because the drug release is dependent on concentration. The Higuchi and Korsmeyer-Peppas models demonstrate robust correlations for both Ginkgo@Niosome and CDOT@Niosome formulations, indicating mechanisms of release that are governed by diffusion. The zero-order model demonstrates a poorer fit, suggesting that the release of the drug is not uniform over time but is influenced by the concentration of the drug present in the system. In a study, they indicated that the optimal model for 5-FU derived from Folic Acid-Decorated pH-responsive Niosomes was the first-order model, as evidenced by an R^2^ value of 0.9695 for this model in relation to 5-FU. This model demonstrates a relationship between the release of the drug and its concentration levels (Rezaei, Rezaei et al. 2022).

Our findings demonstrate that the optimized CDOT@Ginkgo@Niosomes exhibit a particle size of approximately 380 nm (as measured by DLS) and encapsulation efficiencies of 93.22% for CDOT and 98.14% for Ginkgo extract. In comparison, Mourtas et al. reported curcumin-loaded nanoliposomes with mean particle sizes in the range of 150–200 nm and encapsulation efficiencies around 80–85%, achieving a sustained release of approximately 80% over 72 hours (Mourtas, Canovi et al. 2011). Similarly, Agrawal et al. observed that their folic acid-decorated, pH-responsive niosomes presented particle sizes between 150 and 225 nm, with a first-order release kinetic model yielding an R² value of 0.9695 (Agrawal, Tripathi et al. 2017). Our research shows that CDOT provided 39.72% drug release during the first 12 hours whereas Ginkgo released 49.65% of its content at that period before achieving almost complete release (≈99%) at 72 hours. Number-based research indicates that our drug delivery system enhances medication retention along with sustained drug release potential for Alzheimer's disease therapeutic methods.

Figure S2. The release kinetics of Ginkgo@Niosome and CDOT@Niosome formulations using Zero-order, First-order, Higuchi, and Korsmeyer-Peppas models.

**References**

Agrawal, M., D. K. Tripathi, S. Saraf, S. Saraf, S. G. Antimisiaris, S. Mourtas, M. Hammarlund-Udenaes and A. Alexander (2017). "Recent advancements in liposomes targeting strategies to cross blood-brain barrier (BBB) for the treatment of Alzheimer's disease." Journal of controlled release **260**: 61–77.

Mourtas, S., M. Canovi, C. Zona, D. Aurilia, A. Niarakis, B. La Ferla, M. Salmona, F. Nicotra, M. Gobbi and S. G. Antimisiaris (2011). "Curcumin-decorated nanoliposomes with very high affinity for amyloid-β1-42 peptide." Biomaterials **32**(6): 1635–1645.

Rezaei, T., M. Rezaei, S. Karimifard, F. Mahmoudi Beram, M. S. Dakkali, M. Heydari, S. Afshari-Behbahanizadeh, E. Mostafavi, D. O. Bokov, M. J. Ansari, B. Farasati Far, I. Akbarzadeh and C. Chaiyasut (2022). "Folic Acid-Decorated pH-Responsive Nanoniosomes With Enhanced Endocytosis for Breast Cancer Therapy: In Vitro Studies." Frontiers in Pharmacology **13**.
